# circDENND4C, a novel serum marker for epithelial ovarian cancer, acts as a tumor suppressor by downregulating miR-200b/c

**DOI:** 10.1080/07853890.2023.2185289

**Published:** 2023-03-10

**Authors:** Shuang Liu, Limei Yuan, Jinzhu Li, Yurong Liu, Haibo Wang, Xingye Ren

**Affiliations:** a Department of Gynecology, The Fifth People’s Hospital of Jinan, Jinan, China; b Department of Obstetrics, The Fifth People’s Hospital of Jinan, Jinan, China; c Department of Gynecology, The Fourth Peoplés Hospital of Jinan, Jinan, China

**Keywords:** Apoptosis, circDENND4C/miR-200b/c, diagnosis, epithelial ovarian cancer, proliferation

## Abstract

**Research objective:**

To explore the diagnostic value of circ-DENN domain containing 4 C (circDENND4C) in epithelial ovarian cancer (EOC) and the corresponding mechanism.

**Methods:**

We determined the expression of circDENND4C and miR-200b/c in tissues and serum specimens as well as EOC cell lines using qRT-PCR. Basic clinical data, and serum HE4 and CAl25 levels were acquired from patients’ clinical records. Expression-related correlations and the diagnostic value of serum circDENND4C in EOC were also estimated. CCK-8 and flow cytometry were performed to detect the effect of circDENND4C on cell proliferation and apoptosis.

**Results:**

circDENND4C level was lowest while miR-200b/c was highest in EOC tissues, followed by benign and normal tissues. Similarly, serum circDENND4C was lowest while miR-200b/c was highest in EOC patients. Moreover, serum circDENND4C was lower in patients with benign ovarian tumors than in healthy women, while miR-200b/c expression was higher. circDENND4C was negatively associated with miR-200b/c in EOC tissues and serum specimens, and serum circDENND4C was also negatively correlated with serum HE4 and CAl25 in EOC patients. circDENND4C expression in both tissue and serum was negatively related to FIGO and TNM stage, and tumor size in EOC. Serum circDENND4C could distinguish healthy persons from patients with benign ovarian tumors and EOC, and they showed a higher specificity and accuracy than serum CA125 or HE4 in EOC diagnosis. circDENND4C upregulation significantly suppressed EOC cell proliferation and facilitated apoptosis by downregulating miR-200b/c *in vitro*.

**Conclusions:**

Summarily, circDENND4C acts as a tumor inhibitor by downregulating miR-200b/c in EOC and could be a possible tumor marker for EOC diagnosis.

**KEY MESSAGES**
1. circDENND4C expression was lowest while miR-200b/c was highest in EOC tissues or serums, followed by benign and normal tissues or serums.
2. circDENND4C was involved in malignant progression of EOC, concretely, overexpression of circDENND4C suppressed EOC cell proliferation and stimulated apoptosis via downregulating miR-200b/c, and circDENND4C expression in both tissue and serum was closely related to FIGO and TNM stages and tumor size in EOC.
3. Serum circDENND4C showed a higher specificity and accuracy than serum CA125 or HE4 in EOC diagnosis.

**HIGHLIGHTS**
1. circDENND4C expression was lowest while miR-200b/c was highest in EOC tissues, followed by benign and normal tissues.
2. Serum circDENND4C was lowest while miR-200b/c was highest in EOC patients, followed by benign patients and healthy women.
3. Overexpression of circDENND4C suppresses EOC cell proliferation and stimulates apoptosis via downregulating miR-200b/c.
4. circDENND4C expression in both tissue and serum was closely related to FIGO and TNM stage and tumor size in EOC.
5. Serum circDENND4C showed a higher specificity and accuracy than serum CA125 or HE4 in EOC diagnosis.

## Introduction

Ovarian cancer (OC) is one of the most frequent gynecological malignancies, third only to cervical cancer and corpus uteri carcinoma [1]. Epithelial OC (EOC), the most common form of OC, accounts for 90% of all OC cases [2]. EOC has the highest mortality among all gynecologic tumors and poses a serious threat to women’s life [3]. Since ovaries are hidden in the pelvic cavity and no obvious clinical symptoms are observed at early stages, OC is often diagnosed at an advanced stage (III or IV), and the overall prognosis is poor [4]. Although the prognosis of OC has improved dramatically due to advancements in medical technology, the 5-year overall survival rate remains low at about 35–40% [5]. Therefore, early clinical diagnosis is crucial. Commonly used laboratory indicators for OC diagnosis include serum carbohydrate polypeptide antigen 125 (CA125) and serum human epididymal secretory protein 4 (HE4). CA125 is a major OC-related tumor marker that is widely used in clinical settings. It has a high sensitivity but low specificity for the early diagnosis of OC [6]. HE4, discovered in 1999, is a new tumor marker for the diagnosis of OC and has high specificity but low sensitivity [7]. Given their limitations, it is necessary to explore better markers for the early diagnosis of OC.

Circular RNAs (circRNAs), a newly discovered type of endogenous noncoding RNAs [8], are involved in the regulation of various biological functions. They act by sponging off certain microRNAs (miRNAs) and play a role in the pathogenesis of many diseases, including cancers [9–11]. In addition, circRNAs are not commonly degraded by RNA enzymes present in body fluids [8], and most of them possess expression specificity at the tissue, cell, and developmental stage level [12,13]. Thus, they can be ideal clinical biomarkers [14]. So far, the expression and function of circ-DENN domain containing 4 C (circDENND4C) have rarely been studied. Among female cancers, circDENND4C has only been examined in the context of breast cancer (BC). Studies have shown that circDENND4C is overexpressed in BC tissues and cells, and its knockdown can significantly attenuate glycolysis, cellular migration and invasion, and cell proliferation in BC cells under anaerobic conditions [15]. These results indicate that circDENND4C may act as an oncogene in the occurrence and development of BC. Furthermore, circDENND4C expression is associated with TNM stage, lymph node metastasis, and tumor size in BC patients [16], indicating that it may serve as a molecular target for BC diagnosis. Nevertheless, the expression and role of circDENND4C in other gynecological tumors such as OC have not been reported. Moreover, the role of serum circDENND4C as a novel biomarker for the early diagnosis of OC is unclear.

Targeted binding sites between circDENND4C and miR-200b/c have been identified. circDENND4C is known to play a carcinogenic role by negatively regulating miR-200b/c in BC [15]. Increasing evidence demonstrates that miR-200b/c are overexpressed in tissues, cells, whole blood, serum, plasma, and serum-derived exosomes from patients with OC, including EOC, and plays cancer-promoting roles. Thus, it can act as a diagnostic and prognostic biomarker for OC [2,3,17–20]. The combination of serum miR-200a, miR-200b, and miR-200c can distinguish between benign and malignant ovarian tumors [21]. Therefore, we speculated that circDENND4C may be downregulated in OC and regulate the expressions of miR-200b/c, and that serum circDENND4C may be a useful tumor marker for the diagnosis of OC. In this study, we mainly focused on the expression, functional significance, and corresponding molecular mechanism of circDENND4C in EOC.

## Results

### circDENND4C and miR-200b/c expressions and their correlations in tissue and serum samples from EOC patients

**Figure 1.**
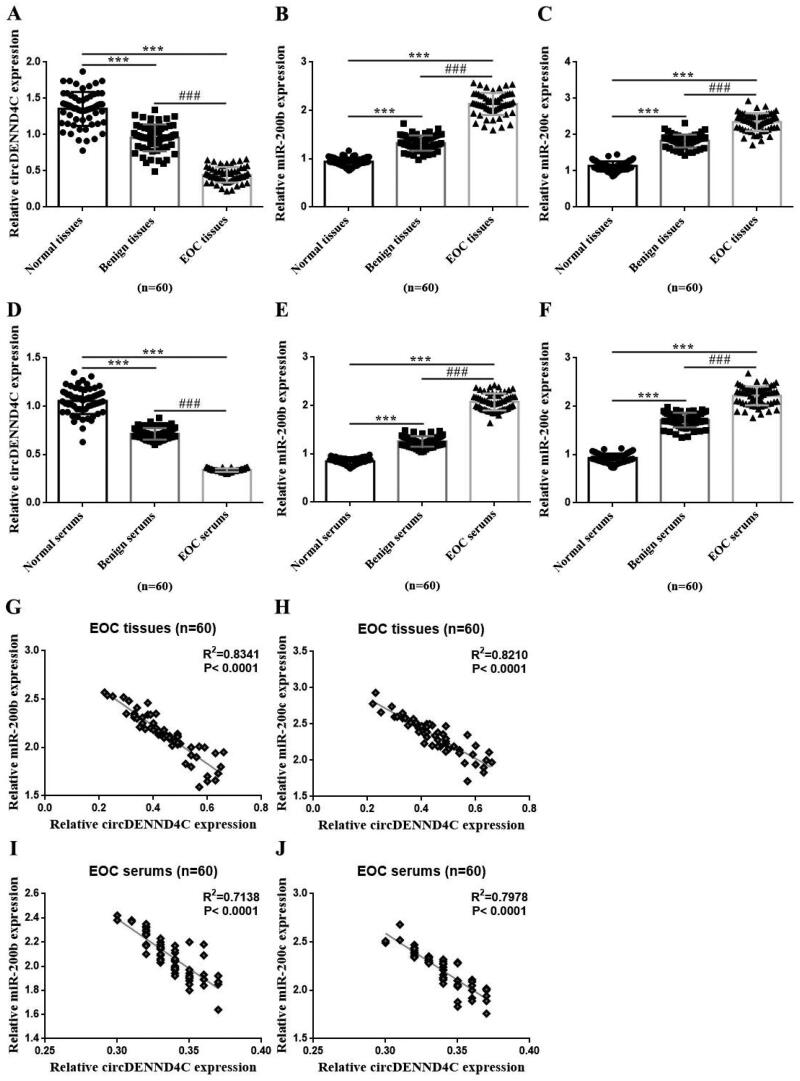
Expression of circDENND4C and miR-200b/c and its correlation in EOC tissue and serum specimens. (a–c) qRT-PCR was performed to determine the relative expression of circDENND4C and miR-200b/c in clinical tissue specimens. (d–f) qRT-PCR was performed to measure the relative expression of circDENND4C and miR-200b/c in clinical serum specimens. (g–j) The correlation between circDENND4C and miR-200b/c expression in EOC tissue and serum specimens. *** vs normal tissue/serum, *p* < 0.001; *^###^* vs benign tissue/serum, *p* < 0.001.

We detected the expression of circDENND4C and miR-200b/c by using qRT-PCR while analysed their expression correlations in those tissue and serum samples from EOC patients with Spearman correlation coefficient analysis. The results were displayed *via* drawing graphs with GraphPad Prism 6.01 software. circDENND4C expression was statistically significant lower while miR-200b/c expression was statistically significant higher in EOC tissues than in benign ovarian tumor tissues and normal paracancerous tissues (2 cm away from EOC tissues and not containing cancer cells by hematoxylin and eosin (HE) staining confirmed by an experienced gynecologic pathologist.) (*p* < 0.001). circDENND4C expression was lower while miR-200b/c expression was higher in benign ovarian tumor tissues than in normal tissues (*p* < 0.001) (Figure 1(a–c)). Similarly, circDENND4C expression was statistically significant lower while miR-200b/c expression was statistically significant higher in serum specimens from EOC patients than in those from patients with benign ovarian tumors and healthy women (*p* < 0.001). Meanwhile, circDENND4C expression was lower while miR-200b/c expression was higher in serum specimens from patients with benign ovarian tumors than in those from healthy women (*p* < 0.001) (Figure 1(d–f)). Interestingly, circDENND4C expression was negatively associated with miR-200b/c expression in both tissue and serum specimens from EOC patients (*p* < 0.0001) (Figure 1(g–j)).

### circDENND4C expression was negatively correlated with HE4/CAl25 levels in the serum of EOC patients

**Figure 2.**
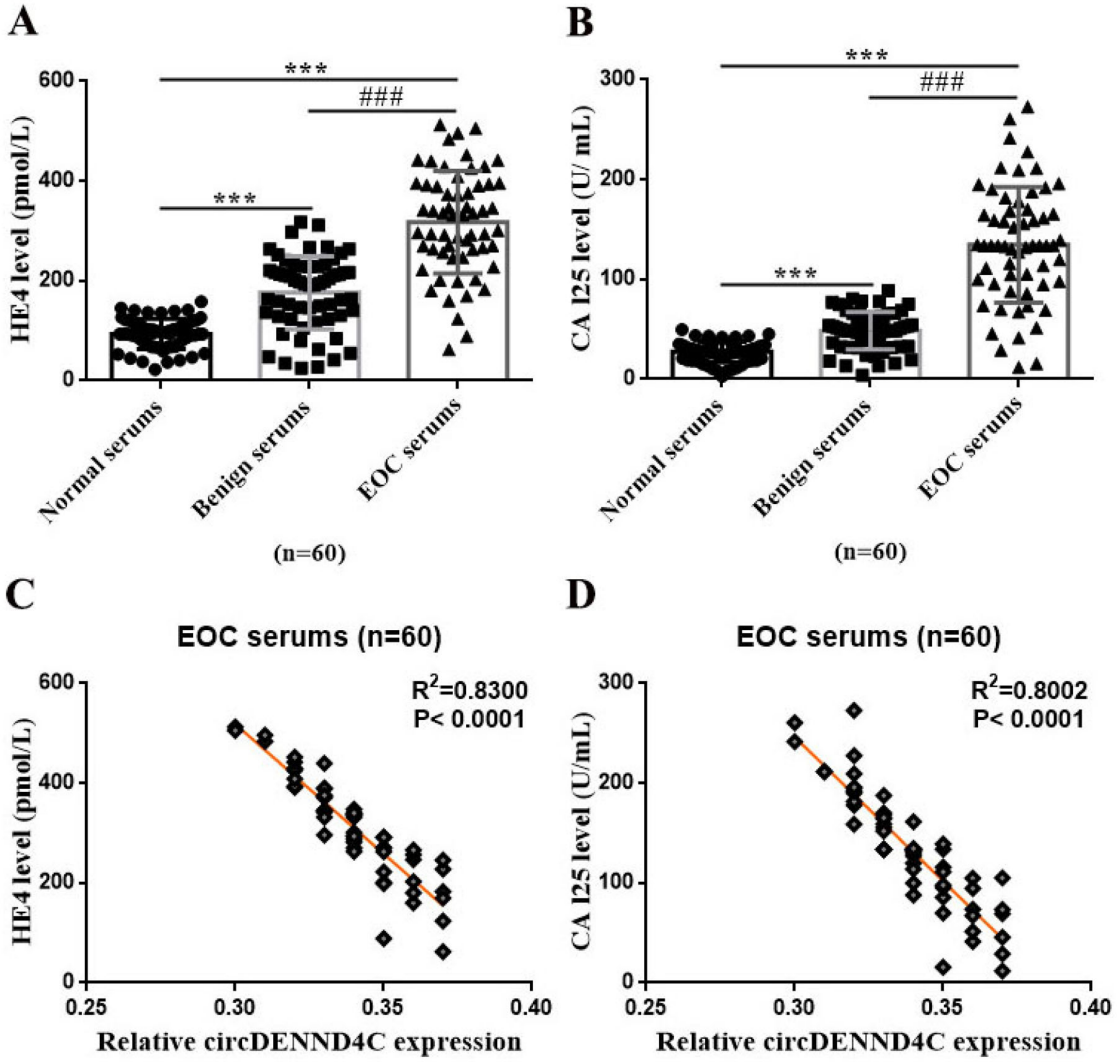
CA125 and HE4 expressions and the correlation between circDENND4C and CA125/HE4 in EOC serum specimens. (a,b) Serum CA125 and HE4 levels in clinical serum specimens. (c,d) The correlation between circDENND4C and CA125/HE4 expression in EOC serum specimens was determined using Spearman correlation coefficient analysis. *** vs normal serum, *p* < 0.001; *^###^* vs benign serum, *p* < 0.001.

As the tumor markers commonly used in clinical laboratory to diagnose OC, serum HE4 and CAl25 levels from the clinical case records revealed that they were the highest in EOC patients, followed by patients with benign ovarian tumors and healthy women (*p* < 0.001) (Figure 2(a,b)). Specifically, serum HE4 and CAl25 levels were significantly higher in EOC patients than in benign ovarian tumor patients and healthy women (*p* < 0.001), and significantly higher in the sufferers with benign ovarian tumor than in healthy women (*p* < 0.001) (Figure 2(a,b)). Interestingly, through a Spearman correlation coefficient analysis, we found that there was a negative association between circDENND4C expression and HE4/CAl25 levels in serum samples from EOC patients (*p* < 0.0001) (Figure 2(c,d)). These findings preliminarily indicated that serum circDENND4C level may be a possible tumor markers for OC diagnosis.

**Figure 3.**
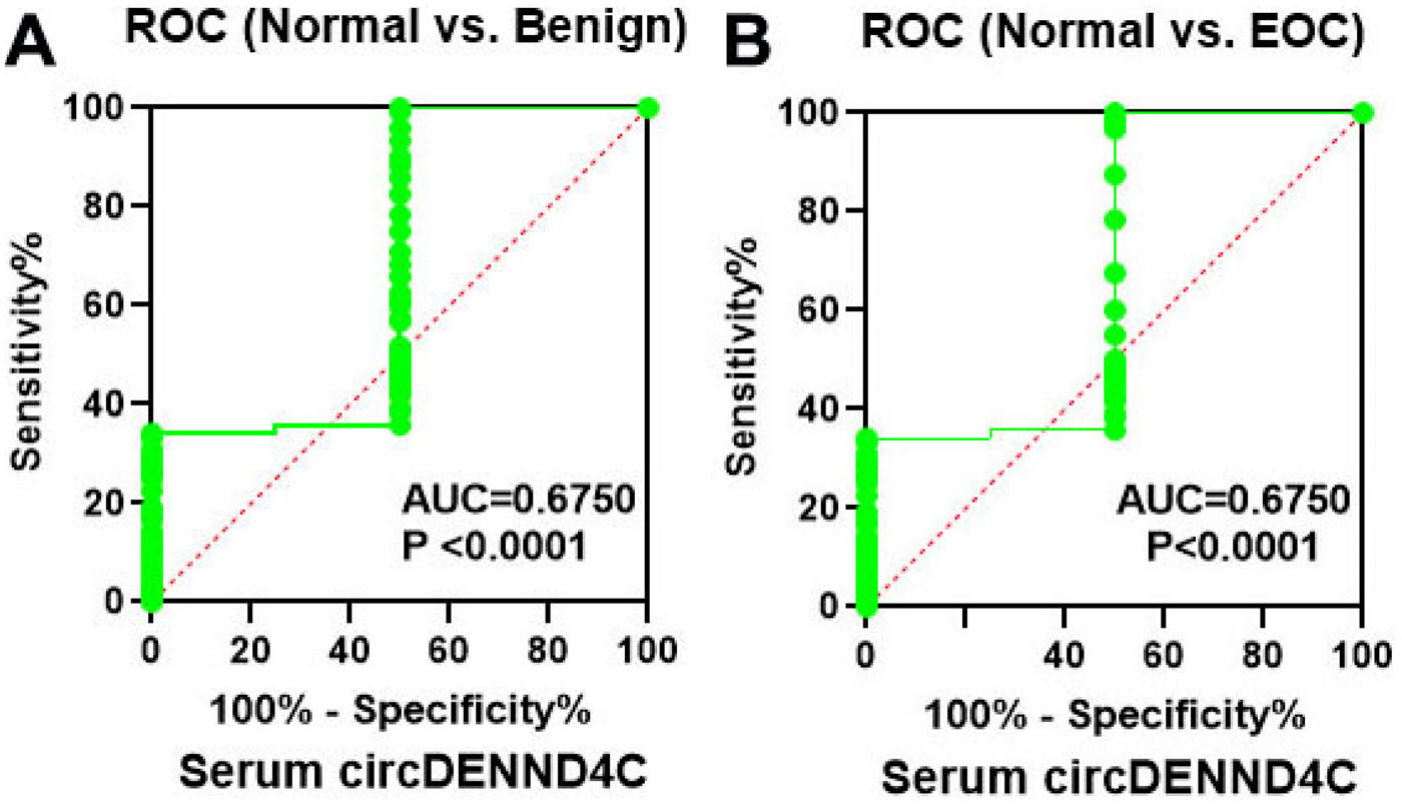
Serum circDENND4C expression differs between patients with benign ovarian tumors/EOC and healthy women. (a,b) ROC analysis shows the sensitivity and specificity of serum circDENND4C in distinguishing patients with benign ovarian tumors/EOC from healthy women. ROC: receiver operating characteristic; AUC: area under the curve.

### Tissue and serum circDENND4C expression was closely associated with disease progression in EOC patients

To analyze the correlations of tissue/serum circDENND4C expression with the clinical features of EOC patients, the patients were divided into low and high circDENND4C expression groups based on median expression values. The outcomes are shown in Table 1. Tissue circDENND4C expression was associated with FIGO stage, TNM stage, histologic grade, tumor size, differentiation degree, and lymph node metastasis (*p* < 0.05/*p* < 0.01), but not with age and menopause status (*p* > 0.05) in EOC patients. Meanwhile, serum circDENND4C expression was correlated with FIGO stage, TNM stage, and tumor size (*p* < 0.05/*p* < 0.01), but not with age, menopause status, histologic grade, differentiation degree, and lymph node metastasis (*p* > 0.05) in EOC patients.

**Table 1.** The expression of circDENND4C in tissues/serums of EOC patients was related to the disease progression.

Parameters	Tissular circDENND4C		*p* Values	Serumal circDENND4C		
	High expression (*n* = 30)	Low expression (*n* = 30)		High expression (*n* = 30)	Low expression (*n* = 30)	*p* Values
Age						
<50	11	13	0.598	10	14	0.292
≥50	19	17		20	16	
Menopause						
Yes	18	16	0.602	19	15	0.297
No	12	14		11	15	
FIGO stage						
I/II	18	8	0.006	17	9	0.030
III/IV	12	22		13	21	
TNM stage						
I/II	19	7	0.001	18	8	0.006
III/IV	11	23		12	22	
Histologic grade						
G1/G2	17	8	0.013	16	9	0.059
G3	13	22		14	21	
Tumor size						
<5 cm	18	9	0.020	18	9	0.020
≥5 cm	12	21		12	21	
Differentiation degree						
Low	8	15	0.032	9	14	0.147
Middle	10	9		10	9	
High	12	6		11	7	
Lymph node metastasis						
Yes	11	20	0.020	12	19	0.071
No	19	10		18	11	

circDENND4C: circ-DENN domain containing 4C; EOC: epithelial ovarian cancer; FIGO: International Federation of Obstetrics and Gynecology; TNM: tumor node metastasis.

### Serum circDENND4C level possessed auxiliary diagnostic value for EOC

Using binary regression analysis and ROC curve analysis, we found that serum circDENND4C level could distinguish healthy persons from those with benign ovarian tumors and EOC (*p* = 0.000) (Table 2 and Figure 3). When serum circDENND4C level was less than 0.7, patients were more likely to have a benign ovarian tumor. When serum circDENND4C expression was less than 0.4, patients were more likely to have EOC (area under the curve [AUC] = 0.675; *p* < 0.0001; 95% confident interval (CI) = 0.6029 − 0.7471) (Table 2). In addition, as shown in Tables 3 and 4, compared with the traditional and well-recognized OC tumor markers (serum CA125 and HE4), serum circDENND4C showed a higher specificity and accuracy (*p* < 0.05 and *p* < 0.01, respectively).

**Table 2.** Analysis of auxiliary diagnostic value of serum circDENND4C in EOC.

Binary regression analysis				
Classifications	Regression coefficient	*p* Value		
Normal vs. benign	−29.144	0.000		
Benign vs. EOC	−151.231	0.994		
Normal vs. EOC	−13.077	0.000		
Normal + benign vs. EOC	−40.112	0.99		

ROC curve analysis				
Classifications	AUC	*p* Value	95% CI	Critical predictive value
Normal vs. benign	0.675	<0.0001	0.6029–0.7471	0.7
Benign vs. EOC	–	–	–	–
Normal vs. EOC	0.675	<0.0001	0.6029–0.7471	0.4
Normal + benign vs. EOC	–	–	–	–

EOC: epithelial ovarian cancer; ROC: receiver operating characteristic; AUC: area under the curve; CI: confident interval.

**Table 3.** Analysis of auxiliary diagnostic results of serum circDENND4C, CA125 and HE4 for epithelial ovarian cancer.

Parameters		Confirmed results (n)		
		Positive	Negative	Total
circDENND4C	Positive	60	5	65
	Negative	0	115	115
CA125	Positive	57	59	116
	Negative	3	61	64
HE4	Positive	57	51	108
	Negative	3	69	72
Total	–	60	120	180

circDENND4C: circ-DENN domain containing 4C; CA125: carbohydrate polypeptide antigen 125; HE4: human epididymal secretory protein 4.

**Table 4.** The diagnostic performance comparison between serum circDENND4C and CA125/HE4 for epithelial ovarian cancer.

Diagnostic markers	Sensitivity (%)	Specificity (%)	Accuracy (%)
Single marker			
CA125^+^	95.00 (57/60)	50.83 (61/120)	65.56 (118/180)
circDENND4C^+^	100.00 (60/60)	95.83 (115/120)	97.22 (175/180)
X^2^	0.039	9.722	6.160
P value	0.844	0.002	0.013
HE4^+^	95.00 (57/60)	57.50 (69/120)	70.00 (126/180)
circDENND4C^+^	100.00 (60/60)	95.83 (115/120)	97.22 (175/180)
X^2^	0.039	6.587	4.368
*p* Value	0.844	0.010	0.037
Combination of two markers			
CA125^+^+HE4^+^	91.67 (55/60)	42.50 (51/120)	58.89 (106/180)
CA125^+^+circDENND4C^+^	95.00 (57/60)	50.00 (60/120)	65.00 (117/180)
X^2^	0.018	0.499	0.335
P value	0.892	0.480	0.563
CA125^+^+HE4^+^	91.67 (55/60)	42.50 (51/120)	58.89 (106/180)
HE4^+^+circDENND4C^+^	95.00 (57/60)	55.83 (67/120)	68.89 (124/180)
X^2^	0.018	1.457	0.860
*p* Value	0.892	0.227	0.354
Combination of two/three markers			
CA125^+^+HE4^+^	91.67 (55/60)	42.50 (51/120)	58.89 (106/180)
CA125^+^+HE4^+^+circDENND4C^+^	91.67 (55/60)	41.67 (50/120)	58.33 (105/180)
X^2^	0.000	0.007	0.003
*p* Value	1.000	0.933	0.956

circDENND4C: circ-DENN domain containing 4C; CA125: carbohydrate polypeptide antigen 125; HE4: human epididymal secretory protein 4.

+: positive.

### Upregulation of circDENND4C inhibited EOC cell proliferation and promoted apoptosis by downregulating miR-200b/c

**Figure 4.**
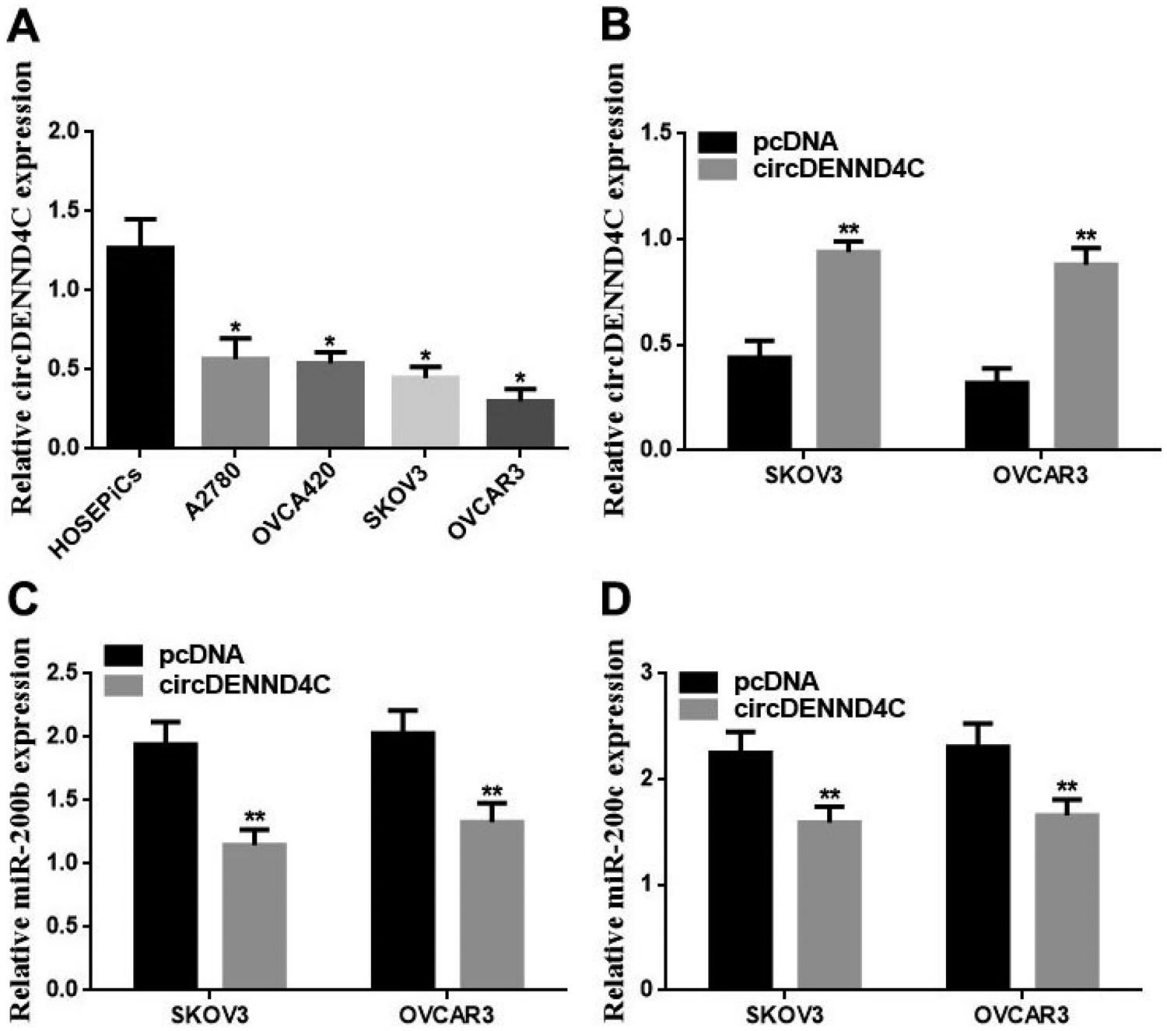
circDENND4C expression and the role on miR-200b/c expression in EOC cells. (a) circDENND4C expression in EOC cells was assessed using qRT-PCR. * vs HOSEPiCs, *p* < 0.05. (b) EOC cells overexpressing circDENND4C were established via the transfection of a pcDNA-based circDENND4C overexpression vector. (c,d) Overexpression of circDENND4C significantly downregulated miR-200b/c expression in EOC cells. ** vs pcDNA, *p* < 0.01.

**Figure 5.**
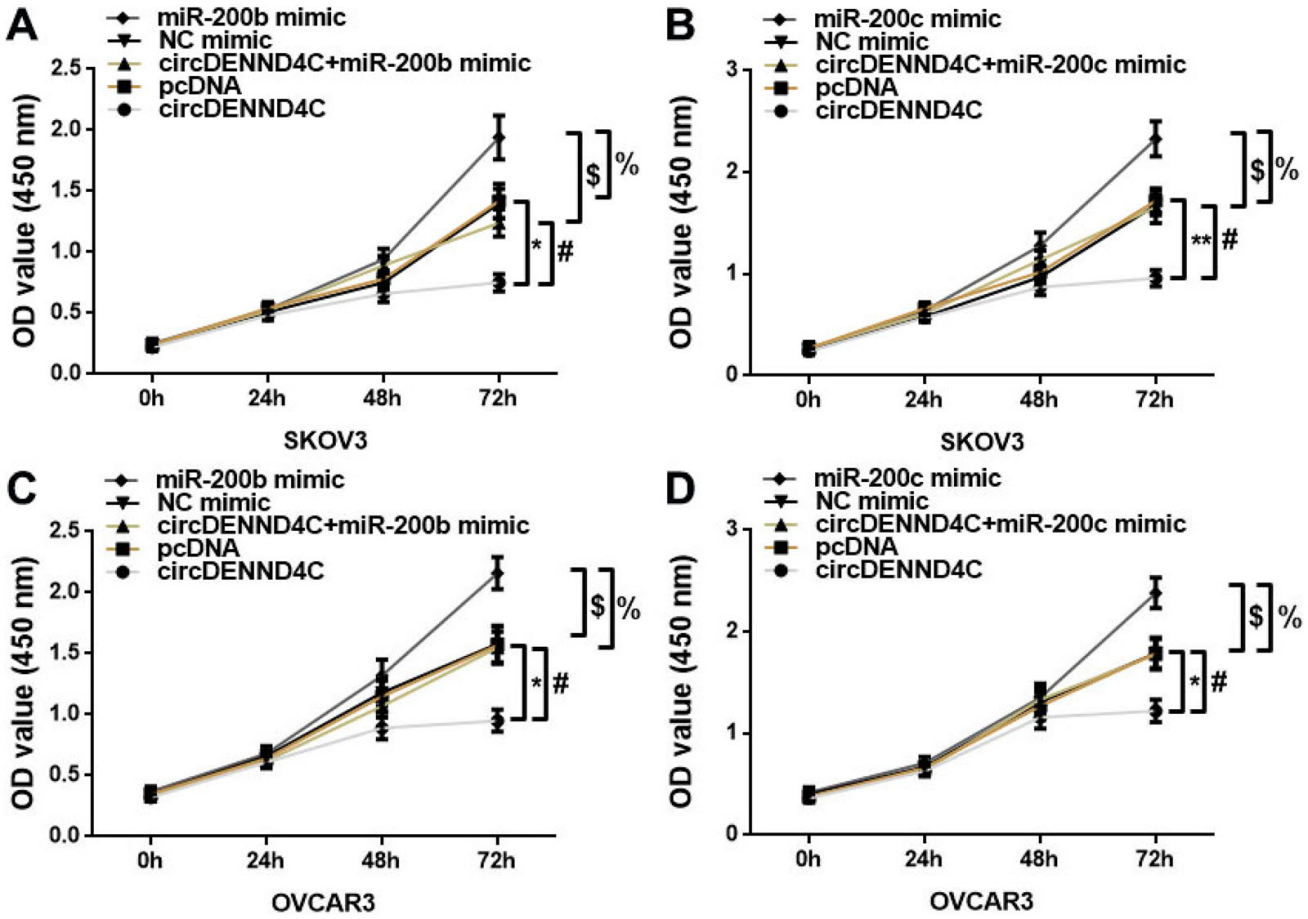
Overexpression of circDENND4C inhibited EOC cell proliferation by regulating the expression of miR-200b/c. (a–d) A CCK-8 proliferation assay was performed to measure the OD value of SKOV3 and OVCAR3 cells at 450 nm and 24 h, 48 h, and 72 h following the transfection of different constructs. * vs pcDNA, *p* < 0.05; ^*#*^ vs circDENND4C, *p* < 0.05; $vs NC mimic, *p* < 0.05; % vs miR-200b/c mimic, *p* < 0.05.

**Figure 6.**
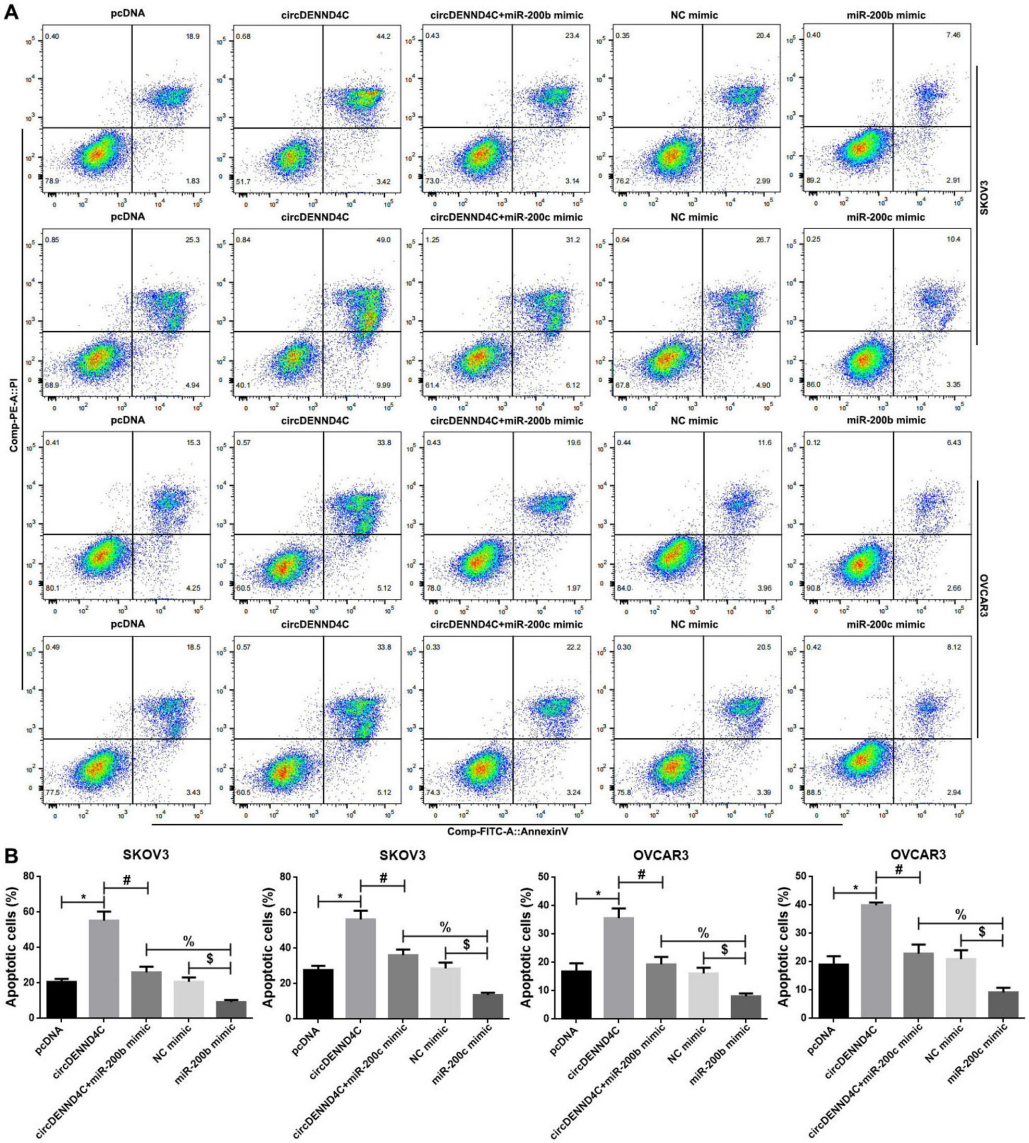
Overexpression of circDENND4C promoted cell apoptosis by regulating the expression of miR-200b/c. (a) Representative images of flow cytometry-based apoptosis analysis. (b) Histogram of results from the flow cytometry apoptosis experiment. * vs pcDNA, *p* < 0.05; ^*#*^ vs circDENND4C, *p* < 0.05; $vs NC mimic, *p* < 0.05; % vs miR-200b/c mimic, *p* < 0.05.

We conducted qRT-PCR to evaluate the circDENND4C expression in a series of human EOC cell lines (OVCA420, OVCAR3, A2780, and SKOV3) and normal human ovarian surface epithelial cells (HOSEPiCs), the results revealed that circDENND4C expression was downregulated in above all the human EOC cells when compared with normal control cells (HOSEPiCs) (*p* < 0.05) (Figure 4(a)). Noteworthily, the lowest circDENND4C expression was detected in OVCAR3 and SKOV3 cells, hence, OVCAR3 and SKOV3 cells were selected for subsequent experiments. Then, to investigate the role and the corresponding mechanism of circDENND4C in EOC, we successfully established circDENND4C overexpressing EOC cells by transfecting a pcDNA-based circDENND4C overexpression vector (circDENND4C) into SKOV3 and OVCAR3 cells in comparison with transfecting a pcDNA empty vector (pcDNA) into these cells (*p* < 0.01) (Figure 4(b)). The overexpression of circDENND4C led to decreases in both miR-200b (*p* < 0.01) (Figure 4(c)) and miR-200c expression (*p* < 0.01) (Figure 4(d)) in SKOV3 and OVCAR3 cells. Finally, CCK-8 and flow cytometry assays were performed to examine cell proliferation and apoptosis in SKOV3 and OVCAR3 cells. The 450 nm OD value at 72 h was lower (*p* < 0.05/*p* < 0.01) and the cell apoptosis rate was higher (*p* < 0.05) in the circDENND4C group than in the pcDNA group in both SKOV3 and OVCAR3 cells. A reverse trend was detected in the circDENND4C + miR-200b/c mimic group when compared with the circDENND4C group (*p* < 0.05). Furthermore, the OD 450-nm value at 72h was higher in the miR-200b/c mimic group than in the NC mimic group (*p* < 0.05/*p* < 0.01), and the apoptosis rate was lower (*p* < 0.05). Interestingly, a reverse trend was discovered in both 450 nm OD value at 72 h and the cell apoptosis rate in the circDENND4C + miR-200b/c mimic group when compared with the miR-200b/c mimic group (*p* < 0.05) (Figures 5 and 6).

## Discussion

To our knowledge, the present study represents the first exploration of the expression, function, and diagnostic value of circDENND4C in patients with gynecological tumors. The results showed that circDENND4C was downregulated in EOC tissues/serum *in vivo* when compared with benign ovarian tumor tissues/serum and normal tissues/serum. It was also downregulated in EOC cells *in vitro*. The overexpression of circDENND4C had an inhibitory effect on cell proliferation and promoted apoptosis in EOC cells *in vitro*. Low circDENND4C expression, both in tissues and serum, was linked with the malignant progression of EOC. Serum circDENND4C levels could distinguish healthy persons from patients with benign ovarian tumors and EOC. Moreover, serum circDENND4C had a better diagnostic performance with a higher specificity and accuracy than CA125 or HE4 levels in the clinical diagnosis of EOC. It has been reported that circRNAs play pivotal roles in human diseases and cancer development and are valuable from a diagnostic standpoint [22,23]. EOC accounts for 90% of all cases of OC, and OC is one of the most common gynecological malignancies [1,2]. Thus, there is a need to seek dependable serum biomarkers for OC and conquer the limitations of imaging. Serum markers such as CAl25 and HE4 are commonly used in clinical settings but have some drawbacks [6,7,24]. A previous study demonstrated that circDENND4C could be a biomarker for the diagnosis of BC given the link between its expression and TNM stage, lymph node metastasis, and tumor size in BC patients [16]. In this study, we first revealed that serum circDENND4C may serve as a novel tumor marker for EOC diagnosis. We found that serum circDENND4C levels were negatively associated with those of validated serum markers CAl25 and HE4 in EOC patients. Moreover, both tissue and serum circDENND4C expression levels were closely related to the FIGO stage, TNM stage, and tumor size in EOC patients.

Increasing evidence demonstrates that circRNAs play a role in the development and progression of cancers by sponging off certain miRNAs [10,11]. miRNAs also play significant modulatory roles in cancers by inhibiting certain genes to negatively regulate messenger RNA (mRNA) expressions [25–27]. A previous study used starBase bioinformatics analysis to predict that there were complementary sites of interaction between circDENND4C and miR-200b/c. The researchers then used luciferase and RIP assays to verify that circDENND4C targets miR-200b and miR-200c in BC [15]. Therefore, in this study, we mainly adopted indirect verification methods to analyze the relationship between circDENND4C and miR-200b/c. We found that miR-200b and miR-200c were upregulated in EOC tissues/serum *in vivo*, and miR-200b/c expression was negatively related to circDENND4C levels in both tissue and serum samples from EOC patients. *In vitro*, the overexpression of circDENND4C resulted in a decrease in miR-200b/c expression in EOC cells. Moreover, the upregulation of miR-200b/c dramatically promoted EOC cell proliferation and inhibited cell apoptosis. Interestingly, it could partially reverse the circDENND4C overexpression-induced decrease in EOC cell proliferation and increase in cell apoptosis. Our finding indicated that miR-200b/c may serve as the target miRNAs of circDENND4C, and circDENND4C may limit cell proliferation and facilitate cell apoptosis by regulating miR-200b/c expression in EOC. The expression of miR-200b and miR-200c is upregulated in tissue, serum, plasma, and whole blood samples from EOC patients and is notably correlated with EOC progression [2,3,28]. Hence, miR-200b/c may be promising candidate biomarkers for EOC diagnosis and could distinguish healthy persons from those with benign tumors and EOC [2,21]. Previous studies have illustrated that miR-200b/c overexpression stimulates the proliferation and inhibits the apoptosis of OC cells, including EOC cells [29,30]. This previous evidence is consistent with our findings in EOC cells. Hence, circDENND4C plays a role in EOC by regulating miR-200b/c expression. However, there was a limitation of this study, the number of enrolled cases is not large, a large sample study may be needed to further confirm our findings.

In conclusion, this study first revealed the novel role of circDENND4C in EOC and highlighted the value of serum circDENND4C as a novel tumor marker for EOC diagnosis. We elucidated that the overexpression of circDENND4C can suppress tumors by inhibiting the proliferation and stimulating the apoptosis of EOC cells *via* miR-200b/c downregulation. The findings put forth a novel serum biomarker for increasing the specificity and accuracy of EOC diagnosis and overcoming the limitations of imaging and traditional serum markers such as CAl25 and HE4. They also demonstrate the value of circDENND4C as a promising therapeutic target for EOC. In the future, a large-scale validation study is warranted.

## Materials and methods

### Ethics statement

This clinical research scheme was authorized by the Ethical Commission of Jinan Fifth Peoplés Hospital and performed in accordance with the Helsinki Declaration (1975) and the International Ethical Guidelines for Biomedical Research Involving Human Subjects (2002). Before specimen collection, written informed consent was provided by all the participants.

### Patients, clinical samples, and data

Overall, 60 EOC (histological subtype: high-grade serous carcinoma (HGSOC)) patients and 60 patients with benign ovarian tumors diagnosed by histopathology at the Jinan Fifth People’s Hospital from January 2020 to December 2021 were included in this study. Moreover, 60 healthy women who participated in physical examination at this center during the same period were enrolled as the control group for only obtaining blood specimens for serum analyses. For patients with EOC and benign ovarian tumors, the inclusion criteria were as follows: (1) No prior treatment with anticancer therapies, such as radiotherapy and chemotherapy before surgery; (2) Absence of other tumors; (3) No dysfunction in organs such as the heart, liver, and kidney; (4) No hematological disease; and (5) Availability of complete clinical case data. EOC tissues with matched normal paracancerous tissues, and benign ovarian tumor tissues were collected from the patients with EOC and benign ovarian tumors, respectively, during surgery and immediately placed in liquid nitrogen. They were then stored in a −80 °C refrigerator for subsequent use. Fasting venous blood specimens were collected at 07:30–10:00 am on admission and immediately sent to the clinical laboratory or placed in liquid nitrogen. They were also stored at −80 °C before serum isolation and subsequent experiments. Serum HE4 and CAl25 levels as well as the basic clinical data including age, menopause state, International Federation of Obstetrics and Gynecology (FIGO) stage, tumor node metastasis (TNM) stage, histologic grade, tumor size, differentiation degree, and lymph node metastasis were collected from the patients’ clinical case records.

### Cell culture and transfection

Normal human ovarian surface epithelial cells (HOSEPiCs) and human EOC cell lines (OVCA420, OVCAR3, A2780, and SKOV3) were provided by the American Type Culture Collection (ATCC) (USA) and cultivated in RPMI-1640 medium (Sigma-Aldrich, R8758) supplemented with 10% fetal bovine serum (FBS) (Gibco, USA) and 1% penicillin/streptomycin (Gibco, BRL) in humidified atmosphere containing 5% CO_2_ at 37 °C. For cell transfection, a pcDNA-based circDENND4C overexpression vector (circDENND4C) and pcDNA empty vector (pcDNA), miR-200b/c mimic, and mimic negative control (NC mimic) were obtained from Genepharma (Shanghai, China). When the confluence reached 60%, the cells were transfected with 200 ng vector or 30 nM oligonucleotides using Lipofectamine 3000 (Invitrogen, Carlsbad, CA, USA) based on the manufacturers’ protocols. After 24 h of transfection, cells were collected for subsequent tests, and the transfection efficiencies were confirmed using qRT-PCR.

### qRT-PCR

qRT-PCR was carried out to detect the expression of circDENND4C and miR-200b/c in clinical tissues, serum specimens, and cells. Based on the manufacturer’s instructions, the TRIzol reagent (Thermo Fisher Scientific, Waltham, MA, USA) was used to isolate RNA from these samples. qRT-PCR was performed with Hifair® III One Step RT-qPCR Probe Kit (11145ES), special primers, and SYBR Green mix (Thermo Fisher Scientific), and the specific qRT-PCR detection steps were in accordance with a previous study [15]. For quantitation, *β-actin* and U6 were utilized as internal controls for circDENND4C and miR-200b/c, respectively, based on the 2−^ΔΔCt^ method.

### Spearman correlation coefficient analysis

For analyzing the correlation between circDENND4C and miR-200b/c expression in EOC tissues and serum and between circDENND4C and HE4/CAl25 expression in serum samples, Spearman correlation coefficient analysis was performed.

### Analysis of the diagnostic value of serum circDENND4C

We adopted two methods for evaluating the diagnostic value of serum circDENND4C. To confirm its value in differential diagnosis, we performed binary regression analysis and receiver operating characteristic (ROC) curve analysis. For confirming its diagnostic performance, the commonly used clinical gold standard was used as a control. The diagnostic thresholds for single serum markers were as follows: circDENND4C ≤ the lowest serum level in normal controls; CA125 ≥ 35 µ/ml; and HE4 ≥ 70 pmol/L (before pausimenia) or HE4 ≥ 140 pmol/L (after pausimenia). For combined detection, if any one tumor marker was positive, the combined test was considered positive. Patients with EOC were classified as positive cases, whereas patients with benign ovarian tumors and healthy women were classified as negative cases. The sensitivity, specificity, and accuracy of each single serum tumor marker for the auxiliary diagnosis of EOC were calculated.

### Cell counting kit-8 (CCK-8) proliferation assay

The proliferation capacities of SKOV3 and OVCAR3 cells transfected with circDENND4C/pcDNA/circDENND4C or miR-200b/c mimic/NC mimic/miR-200b/c mimic were evaluated using the CCK-8 reagent (ACMEC Biochemical, Shanghai, China) based on the manufacturer’s instructions. For quantification, optical density (OD) values at 450 nm were recorded at 24 h, 48 h, and 72 h on a microplate reader (Bio-Rad, Hercules, CA, USA).

### Flow cytometry analysis

Above SKOV3 and OVCAR3 cells transfected with different constructs were examined for apoptosis using a flow cytometry assay with the fluorescein isothiocyanate (FITC) Annexin-V Apoptosis Detection Kit I (BD Pharmingen, San Diego, CA, USA) based on the manufacturer’s guidelines. A FACS Canto II flow cytometer (BD Biosciences) was employed to measure apoptosis rates.

### Statistical analysis

SPSS software (version 21.0, Inc., Chicago, IL, USA) was adopted for statistical analysis, and the outcomes were represented as the mean ± standard deviation (SD) or number (*n*) and rate (%). Quantitative data were compared among multiple groups (more than two) using one-way analysis of variance (ANOVA). Categorical data were compared between groups using the X^2^ test, while ranked data were analyzed using the Pearson X^2^ test or non-parametric tests (Somers’ D/Kendall’s tau-b). The Spearman correlation coefficient analysis method was employed to analyze expression-related correlations. *p*-Values < 0.05 were regarded as statistically significant.

## Data Availability

All the data produced or analyzed in the present work are available from the corresponding author on reasonable request.
